# Efficacy of spinal fusion in Brucella spondylitis: a systematic review

**DOI:** 10.3389/fsurg.2025.1537153

**Published:** 2025-04-25

**Authors:** Kai He, Wenhua Xing

**Affiliations:** ^1^ Inner Mongolia Medical University, Hohhot, Inner Mongolia Autonomous Region, China; ^2^ The Second Affiliated Hospital of Inner Mongolia Medical University, Hohhot, Inner Mongolia Autonomous Region, China

**Keywords:** Brucella spondylitis (BS), systematic evaluation, spinal fusion, biofilm, bacterial inflammation

## Abstract

**Background:**

Brucellosis affects more than 500,000 people worldwide each year, and brucellosis spondylitis is one of its most common complications, accounting for about 2–53 percent of cases.

**Purpose:**

The aim of this study was to systematically review the literature on the outcome of spinal fusion in patients with Borrelia burgdorferi spondylitis.

**Study design:**

Systematic review.

**Methods:**

Seventeen studies including 746 patients treated with minimally invasive and/or open spinal fusion were reviewed. Patients with this pathologic spondylolisthesis showed significant improvement in clinical and functional scores and high satisfaction after spinal fusion. The overall fusion rate was 99%, the excellent fusion rate was 90%, and all patients with fusion devices placed achieved fusion. The Oswestry disability index scores decreased from 62.11 (35.72–88.5) preoperatively to 7.22 (4.0–10.44) postoperatively, the Japanese Orthopaedic Association scores improved from 15.13 (range, 10.47–19.79) preoperatively to 22.17 (16.4–27.93) postoperatively, and the The visual analog scale scores improved from 6.95 (4.5–9.4) to 1.33 (0–2.66) postoperatively, Erythrocyte sedimentation rate from 49.95 (35.5–64.4) preoperatively to 8.17 (4.13–12.2) postoperatively, and C-reactive protein from 41.25 (20.3–62.2) preoperatively to 4.48 (1.25–12.2) postoperatively. In addition, all patients showed varying degrees of neurological improvement, with a probability of complete return to normal neurological symptoms of 88.79%. Spinal deformities also improved significantly.

**Conclusion:**

Spinal fusion can achieve a high clinical success rate and has a favourable prognosis and pain relief in patients with Brucella spondylitis. Although patients with Brucella spondylitis have a number of high-risk factors affecting the outcome of fusion, in conjunction with medication and debridement, spinal fusion may be a good option with significant functional and clinical improvement.

## Introduction

The prevalence of brucellosis is very high, with more than 500,000 people suffering from it annually worldwide (1), and brucellosis spondylitis is one of its most common complications, accounting for approximately between 2% and 53% (2). Skeletal Brucella infections typically exhibit a variety of risk factors, including decreased bone quality at the site of infection, disturbances in bone metabolic homeostasis, biofilm formation from implants, and the patient's own nutritional deficiencies, which may preclude spinal fusion and may make clinical outcomes more difficult to predict (3, 4).

Patients with Brucella spondylitis have a strong desire for relief of back pain and restoration of mobility and neurological function. Surgery is often required once conservative treatment has failed for 3 months, or when a paravertebral abscess is triggered, neurological function is impaired, and back pain that severely affects quality of life develops (5). Although spinal fusion has been practised in Brucella spondylitis over the past 20 years, it is doubtful whether spinal fusion is feasible in spines affected by inflammation. Several studies have documented better postoperative functional gains and fusion of spinal structures. Achieving pain relief, improved neurological function, and restoration of spinal stability can reasonably be considered one of the main goals of fusion surgery (4, 6–21). Since in Brucella spondylitis is a pathological inflammatory response to bacterial infection, there are many risk factors affecting fusion, and experts have been debating whether spinal fusion can be applied to this population. There is little survey data and no available treatment guidelines for patients with Brucella spondylitis.

Based on several small case series reporting successful clinical outcomes in patients with Brucella spondylitis after spinal fusion surgery (4, 6–21), this systematic review aims to summarise these findings and assess the clinical outcomes of spinal fusion in patients with Brucella spondylitis. In doing so, we aimed to discover whether spinal fusion surgical treatment facilitates spinal fusion and good clinical outcomes in patients with Brucella spondylitis who meet the surgical indications for it to be an important treatment option.

## Methods

This systematic evaluation was conducted in accordance with the PRISMA (Preferred Reporting Items for Systematic Reviews and Meta-Analyses) statement guidelines (22).

## Literature search strategy

A comprehensive search strategy was developed using the Cochrane Collaboration guidelines for the following scientific electronic databases: Pubmed, Medline, Embase and the Cochrane Library. The final search date was 28 September 2024. We used the key search terms “Brucellosis”, “Spondylitis” and “Spinal Fusion” to identify all relevant studies. The search was limited to results from four electronic databases: Pubmed, Medline, Embase and the Cochrane Library. Initial screening of titles and abstracts was carried out using two independent observers, followed by a review of the full text of the selected papers.

## Evaluation of the study quality

The methodological quality of the study was independently assessed by each author using a 10-item Coleman Methodology Score (CMS), which classifies articles as excellent (85–100), good (70–84), fair (55–69), and poor (<55) based on the total score.

## Selection criteria

We included articles reporting outcomes after minimally invasive and/or open treatment of brucellosis spondylitis. These articles had to report the outcome of interest, including type of surgery, fusion outcome, mean follow-up time and time to fusion, patient-reported outcome measures, and postoperative complications. We excluded conference abstracts, surgical techniques, reviews, clinical commentaries, and non-peer-reviewed papers. There were no restrictions on gender, time since surgery, recruitment methods or rehabilitation programmes. Two evaluators independently applied the eligibility selection criteria to articles identified during the database search by reviewing titles and abstracts. When it was unclear after such a review whether a study was suitable for inclusion, the full text was assessed and cross-checked for eligibility. Disagreements between evaluators were resolved by consensus, and a third evaluator was consulted when consensus could not be reached.

## Data extraction and synthesis

Two independent evaluators extracted information from the included studies. Study characteristics, patient demographics, diagnostics (imaging, epidemiological exposure history, laboratory tests), lesion site, type of procedure, clinical and imaging follow-up intervals, complications, and clinical and imaging outcomes were extracted and recorded. The various imaging metrics included were total number of fusions, number of 1-level fusions, number of 2-level fusions, time to fusion, and presence or absence of interbody fusion devices. The various clinical outcomes included were ASIA (American Spinal Injury Association) VAS (visual Analogue Scale), ODI (Oswestry Disability Index), JOA (Japanese Orthopaedic Association), ESR (Erythrocyte Sedimentation Rate), CRP (c-reactive protein), cobb angles, and NDI (Cervical Disability Index), with ASIA graded A-E (17): (A) no sensory or motor function; (B) incomplete sensory but no motor function; (C) incomplete motor function is preserved below the neurological level and more than half of the key muscles below the neurological level have a muscle grade less than 3; (D) incomplete motor function is preserved below the neurological level, and more than half of the key muscles below the neurological level have a muscle grade greater than or equal to 3; and (E) sensory and motor function are normal. Among the spinal fusion grades according to Bridwell's grading of 1–4 (23), Grade 1: complete healing fusion; Grade 2: mostly healing fusion; Grade 3: partially healing fusion; Grade 4: poorly healing not fused, the total fusion includes Grade 1 and Grade 2 fusion, and Grade 1 is good fusion. Subsequently, the characteristics and results of all eligible studies were combined. Results presented inconsistent characteristics between articles, and results were presented in the form of narrative descriptions.

## Results

### Study selection

**Figure 1:**
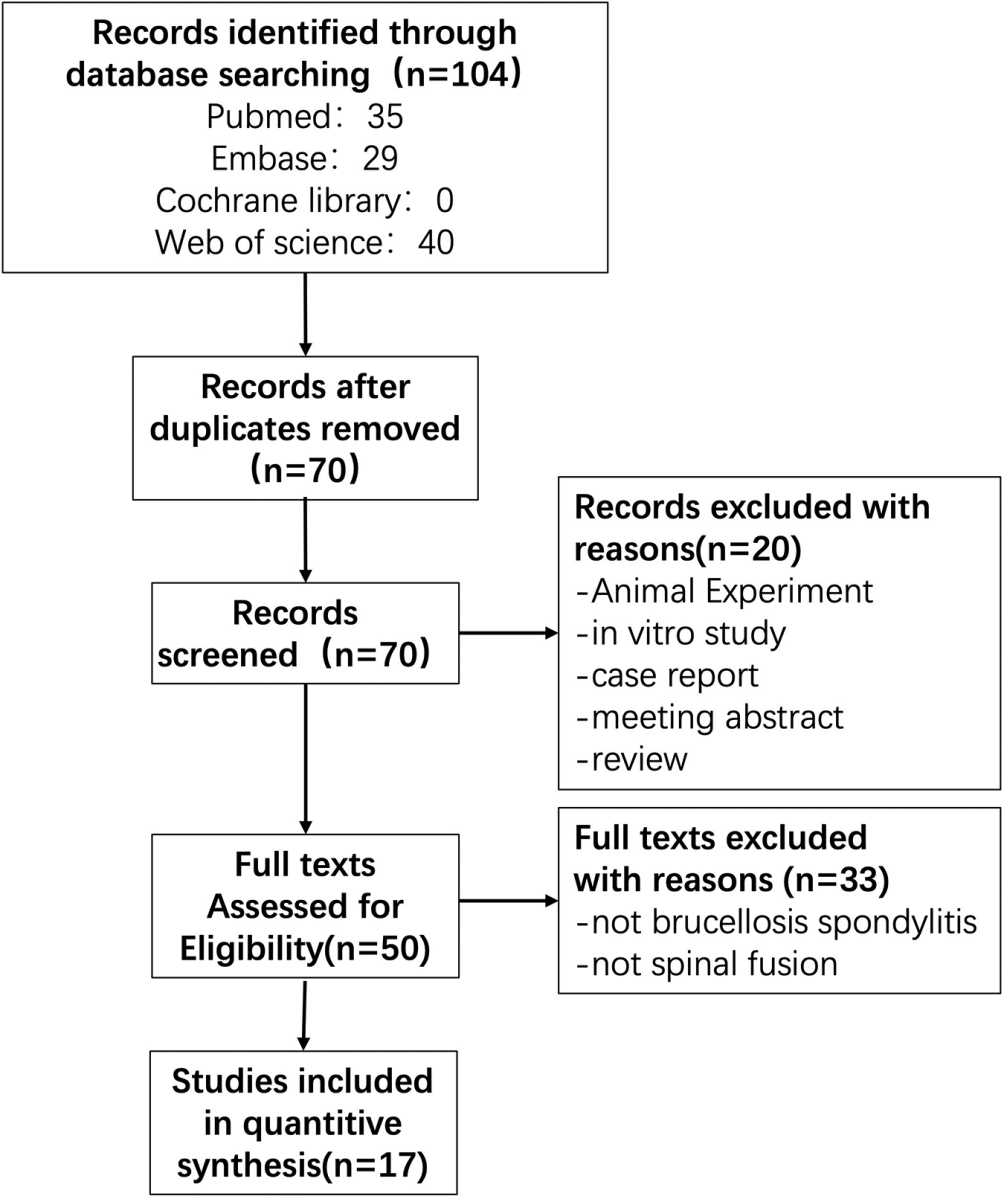
The PRISMA (preferred reporting items for systematic meta-analyses) flow diagram.

We initially identified 104 articles for evaluation based on the search strategy described above. We excluded 44 duplicates, leaving 70 articles for title and abstract browsing. 20 articles were excluded because they were animal studies, *in vitro* studies, case reports, conference abstracts, and review articles. A comprehensive review of the remaining articles and their citations, as well as a detailed search of the literature, excluded 33 non-Brucella spondylitis, non-spinal fusion studies, and ultimately 17 studies (4, 6–21) were finally included in the current systematic evaluation (Figure 1).

### Study characteristics and quality

An Excel spreadsheet (Microsoft) was developed to summarise the data from all studies. The characteristics of the included studies are shown in Table 1. Table 2 lists the demographics of the available patient cohorts, lesion sites, and surgical techniques used in each study.

**Table 1:** Study characteristics.

Study	Year	Design	Level of evidence	Mean follow-up (range), mo	Coleman score
Wang et al.	(7)	Retrospective	4	13.92 ± 1.5	55
Wang et al.	(16)	Retrospective	4	3, 6, 9	52
JIA et al.	(10)	Retrospective	4	25.76 ± 5.81	68
Zhang et al.	(6)	Retrospective	4	25.0 ± 8.1	58
Zhang et al.	(8)	Retrospective	4	17.9 ± 5.2	55
Jiang et al.	(12)	Retrospective	4	25.4 ± 1.5	68
Abulizi et al.	(13)	Prospective	3	24.9 ± 8.2	77
Li et al.	(14)	Retrospective	4	>12	62
Wang et al.	(17)	Retrospective	4	16.8 ± 4.2	55
Luan et al.	(18)	Retrospective	4	31.2 ± 9.6	65
Yin et al.	(20)	Retrospective	4	35.3 ± 8.1	54
Chen et al.	(19)	Retrospective	4	14.3 ± 3.5	56
Zhao et al.	(4)	Retrospective	4	14.45 ± 4.25	64
Na et al.	(21)	Retrospective	4	Anterior group: 31.6 ± 6.3	62
				Posterior group: 32.8 ± 4.8	
Liu et al.	(9)	Retrospective	4	14.8	59
Yang et al.	(11)	Retrospective	4	25	69
Su et al.	(15)	Retrospective	4	20.2	58

**Table 2:** Patient demographics.

Study	Year	Spine (patients), *n*	Male: female, *n*	Age, mean ± SD (range), y	Mean follow-up (range), mo	Operation type	Location Segment: patients, *n*
Wang et al.	(7)	13	10:3	52 ± 9.77	13.92 ± 1.5	ULIF	13 one segment (1 L1–2, 1 L2–3, 2 L3–4, 4 L4–5, 5 L5–S1)
Wang et al.	(16)	22	17:5	53.9 ± 9.9	NA	20 TLIF, 1 anterior combined with posterior surgery, 1 revision surgery	16 one segment(4 L2–3, 3 L3–4, 3 L4–5, 6 L5-S1), 6 over one segment(1 L3–5, 4 L4–S1, 1 L2–3 + L4–S1)
Jia et al.	(10)	80	15:41	PLIF group: 54.3 ± 5.8/OLIF group: 54.5 ± 5.4	25.76 ± 5.81	40 PLIF, 40 OLIF	61 one segment (21 L3–4, 29 L4–5, 11 L5–S1), 19 over one segment (8 L2–4, 11 L3–5)
Zhang et al.	(6)	16	14:2	59.2 ± 6.5	25.0 ± 8.1	Posterior surgery	16 one segment (3 L1–2, 2 L2–3, 1 L3–4, 4 L4–5, 6 L5–S1)
Zhang et al.	(8)	15	9:6	55.5 ± 8.4	17.9 ± 5.2	Anterior surgery	14 one segment (1 C3–4, 4 C4–5, 4 C5–6, 5 C6–7), 1 over one segment (1 C4–5 + C6–7)
Jiang et al.	(12)	62	29:36	Posterior group: 44.6 ± 13.5/anterior combined with posterior group: 46.3 ± 14.9	25.4 ± 1.5	33 posterior surgey, 29 anterior combined with posterior surgery	NA
Abulizi et al.	(13)	32	NA	NA	24.9 ± 8.2	TLIF	NA
Li et al.	(14)	63	48:15	Non-debridement group:55.5 ± 10.3/debridement group: 51.2 ± 10.0	NA	34 non-debridement surgery, 29 debridementsurgery	63 one segment (7 T12–L1, 5 L1–2, 10 L2–3, 15 L3–4, 15 L4–5, 11 L5–S1)
Wang et al.	(17)	14	8:6	49.1 ± 8.0	16.8 ± 4.2	OLIF	14 one segment (1 L1–2, 2 L2–3, 8 L3–4, 3 L4–5)
Luan et al.	(18)	55	40:15	39.8 ± 14.7	2.6 ± 0.8y	Posterior surgery	34 one segment (3 T12, 1 L1–2, 7 L2–3, 3 L3,1 L3–4, 8 L4–5, 2 L5, 1 S1, 7 L5–S1, 1 S1–2), 21 over one segment(1 T10–L2, 1 T11–L2, 5 T12–L2, 1 T12–L3, 2 L1–3, 1 L1–4, 4 L2–4, 6 L3–5)
Yin et al.	(20)	16	12:4	45.0 ± 10.3	35.3 ± 8.1	Anterior surgery	14 one segment (1 L1–2, 2 L2–3, 5 L3–4, 3 L4–5, 3 L5–S1), 2 over one segment (1 L1–3, 1 L2–4)
Chen et al.	(19)	24	11:13	56.1 ± 10.7	14.3 ± 3.5	Posterior surgery	22 one segment (2 L1–2, 3 L2–3, 3 L3–4, 8 L4–5, 6 L5–S1), 2 over one segment(1 L1–3, 1 L1–2 + L5–S1)
Zhao et al.	(4)	61	42:19	56.33 ± 9.16	14.45 ± 4.25	Posterior surgery	49 one segment (2 T12–L1, 5 L1–2, 11 L2–3, 13 L3–4, 12 L4–5, 6 L5–S1), 12 over one segment (1 T12–L2, 1 L1–3, 1 L2–4, 1 L3–5, 1 T12–L3, 1 L2–5, 1 L1–5, 1 L4–5 + T12–L1, 1 L4–5 + L1–2, 2 L4–5 + L2–3, 1 L3–4 + T7–8)
Na et al.	(21)	27	9:18	Anterior group: 39.8 ± 12.2/posterior group: 43.5 ± 11.3	Anterior group: 31.6 ± 6.3/posterior group: 32.8 ± 4.8	14 anterior surgery, 13 posterior surgery	NA
Liu et al.	(9)	32	20:12	ULIF group: 51.7 ± 12.0/posterior group: 53.1 ± 9.3	14.8	15 ULIF, 17 posterior surgery	32 one segment (3 L1–2, 3 L2–3, 6 L3–4, 13 L4–5, 7 L5–S1)
Yang et al.	(11)	148	80:68	Posterior group: 46/anterior group: 44	25	78 posterior surgery, 70 anterior surgery	125 one segment (1 T10–11, 2 T12–L1, 6 L1–2, 62 L3–4, 49 L4–5, 5 L5–S1), 23 over one segment (2 T8–10, 9 L2–4, 12 L3–5)
Su et al.	(15)	28	NA	NA	20.2	12 TLIF, 16 anterior surgery combined with posterior surgery	28 one segment (1 T1–T2, 2 L1–2, 5 L2–3, 11 L3–4, 7 L4–5, 2 L5–S1)

ULIF, unilateral biportal endoscopic lumbar interbody fusion; TLIF, transforaminal lumbar interbody fusion; OLIF, oblique lateral interbody fusion; PLIF, posterior lumbar interbody fusion; NA, not available.

Quality assessment and bias analysis of the included articles were performed using Coleman methodological scores; fourteen studies, fair; one study, good; and two studies, poor. The quality score of the articles was the average of the scores of the two researchers, which was 61 (range, 52–77). The level of evidence of the included articles ranged between III and IV, with one study being level IV evidence (13) and remainder sixteen studies being level III evidence and (Table 1).

### Patient demographics

All studies reported clinical outcomes associated with spinal fusion for Brucella spondylitis. Seventeen studies included a total of 746 patients, with a mean age of 49.5 (range, 38.8–59.2) years (Table 2). Twelve studies (4, 6–13, 16, 17, 19) reported the imaging method used to confirm the diagnosis, with a total of 519 patients in whom x-ray, CT, and MRI were used.All studies reported the laboratory tests used to confirm the diagnosis, with every study included 2 or more laboratory tests, the most used was SAT with fourteen studies (4, 7, 9–16, 18–21), followed by BC with seven studies (4, 7, 8, 12, 13, 18, 20) and PBPT with seven studies (6, 8–11, 14, 19). Five studies (6, 9, 11, 14, 16) reported history of epidemiological exposure, a total of 246 patients out of 248 (99.19%) had a history of epidemiological exposure. Ten studies (4, 6–8, 13, 16–18, 20, 21) included 285 reported clinical symptoms, localised pain in the spine was reported by 270 (94.74%); radicular limb pain in 120 (42.11%); fever in 185 (64.91%); sweating in 94 (32.98%); weakness and loss of appetite in 98 (34.39%); loss of body weight in 75 (26.32%); and arthralgia in 29 (10.18%); and there were other rare complications, the such as hepatomegaly, splenomegaly, myalgia and testicular pain (Table 3).

**Table 3:** Diagnostics.

Study	Year	Spines(patients), *n*	Spines with imaging, *n*	Imaging	Patients with laboratory tests, *n*	Laboratory tests	Patients with epidemiological exposure history, *n*	Patients with symptoms
Wang et al.	(7)	13	13	x-ray, CT, MRI	13	BC, BC-BS, SAT	NA	13 Back pain, 11 Lower limb neurogenic symptoms, 3 Fever, 4 Sweating, 13 Loss of appetite or weakness or fatigue, 7 Weight loss, 2 Arthralgia
Wang et al.	(16)	22	22	x-ray, CT, MRI	22	SAT, Coombs, ICAT	22	22 Back pain, 19 Lower limb pain, 18 Fever, 10 Sweating
Jia et al.	(10)	80	80	x-ray, CT, MRI	80	SAT, Coombs, RBPT, CFT	NA	NA
Zhang et al.	(6)	16	16	x-ray, CT, MRI	16	RBPT, BC-BS	14	16 Back pain, 10 Lower limb neurogenic symptoms, 12 Fever, 12 Sweating, 14 Loss of appetite or weakness or fatigue, 10 Weight loss
Zhang et al.	(8)	15	15	x-ray, CT, MRI	15	RBPT, STAT, BC, BC-BS	NA	15 Neck pain, 7 Upper limb neurogenic symptoms, 6 Fever, 9 Loss of appetite or weakness or fatigue, 7 Weight loss or Weight loss
Jiang et al.	(12)	62	62	x-ray, CT, MRI	62	BC, SAT	NA	NA
Abulizi et al.	(13)	32	32	x-ray, CT, MRI	32	BC, SAT	NA	31 Back pain, 22 Lower limb neurogenic symptoms, 27 Fever, 18 Sweating, 14 Loss of appetite or weakness or fatigue, 9 Weight loss, 4 Arthralgia, 7 Hepatomegaly
Li et al.	(14)	63	63	NA	63	SAT, RBPT	32	NA
Wang et al.	(17)	14	14	x-ray, CT, MRI	14	NA	NA	14 Back pain, 6 Lower limb neurogenic symptoms, 3 Fever
Luan et al.	(18)	55	55	NA	55	BC, SAT	NA	55 Back pain, 28 Lower limb neurogenic symptoms, 41 Fever
Yin et al.	(20)	16	16	NA	16	BC, BC-BS, SAT	NA	16 Back pain, 16 Loss of appetite, 15 Fever, 12 Weight loss
Chen et al.	(19)	24	24	x-ray, CT, MRI	24	SAT, RBPT	NA	NA
Zhao et al.	(4)	61	61	x-ray, CT, MRI	61	BC, SAT	NA	61 Back pain, 54 Fever, 50 Sweating, 32 Loss of weakness or fatigue, 30 Weight loss, 23 Arthralgia, 13 Myalgia, 4 Splenomegaly, 2 Hepatomegaly, 2 Testicular pain
Na et al.	(21)	27	27	NA	27	BC-BS, SAT	NA	27 Back pain, 14 Lower limb neurogenic symptoms, 12 Fever
Liu et al.	(9)	32	32	x-ray, CT, MRI	32	SAT, RBPT	32	NA
Yang et al.	(11)	148	148	x-ray, CT, MRI	148	BC, SAT, Coombs, RBPT, CFT	148	NA
Su et al.	(15)	28	28	NA	28	SAT	NA	NA

CT, computed tomography; MRI, magnetic resonance imaging; BC, blood culture; BC-BS, brucellosis of biopsy specimens; SAT, serum agglutination test; ICAT, immune-capture agglutination test; RBPT, rose Bengal plate test; CFT, complement fixation test; STAT, standard tube agglutination test; TLIF, transforaminal lumbar interbody fusion; OLIF, oblique lateral interbody fusion; PLIF, posterior lumbar interbody fusion; NA, not available.

### Site of the lesion and treatment

Fourteen studies (4, 6–11, 14–20) reported the involvement of segments in Brucella spondylitis, including a total of 587 individuals (Table 2). Of these, 501 (85.35%) had single segment involvement, 86 (14.65%) had multiple segment involvement, the commonly involved segments were L3–4 with 208 (35.43%), L4–5 with 185 (31.52%), L5–S1 with 75, and the remaining other sites were less commonly seen. Seven studies (7, 9, 10, 13, 15–17) reported the specific surgical approach, two studies (7, 9) used ULIF, three studies (13, 15, 16) used TLIF, two studies (13, 17) used OLIF, and one study (10) used PLIF, and all studies reported the direction of approach, fourteen studies (4, 6, 7, 9–16, 18, 19, 21) used posterior approach, five studies (8, 11, 17, 20, 21) used an anterior approach, and three studies (12, 15, 16) used combined anterior and posterior approach, and in addition, one study (14) reported cleared and un-cleared approaches.

### Clinical and functional scores

Studies that processed the required data explored statistical significance by comparing preoperative and postoperative outcome scores (Table 4). Fourteen studies (6–19) assessed ESR from 49.95–9.17, twelve studies (6–9, 12–19) reported CRP from 41.25–4.48, six studies (7, 10, 11, 14, 15, 21) reported cobb angles from 30.33–23.92, all studies reported VAS from 6.95–1.33, seven studies (7–9, 12–14, 18) reported JOA from 15.13–22.17, nine studies (6, 7, 9, 12, 13, 15–18) reported ODI from 62.11–7.22. One study (8) reported NDI from 25–3.7, and ten studies (4, 6, 7, 10, 11, 15, 17–20) reported ASIA. All patients showed varying degrees of improvement in neurological functioning, and 198 out of 223 patients with neurological impairments showed a complete return to normal neurological function. The complete recovery rate was 88.79%.

**Table 4:** Outcome scores.

Study	Year	Outcome scores	Preoperative scores	Postoperative scores	Increase points	Outcome category	Preoperative category, n	Postoperative category, *n*
Wang et al.	(7)	ESR, CRP, cobb angles, VAS (back), VAS (leg), JOA, ODI	38.69 ± 18.98, 26.82 ± 19.87, 47.18 ± 6.88, 5.85 ± 1.28, 3.69 ± 2.02, 13.46 ± 3.18, 55.57 ± 10.99	5.92 ± 2.81, 4.25 ± 1.91, 42.26 ± 6.92, 0.38 ± 0.51, 0.23 ± 0.44, 27.08 ± 0.95, 6.14 ± 3.38	32.77, 22.57, 4.92, 5.47, 3.46, 13.62, 49.43	ASIA	5D, 8E	13E
Wang et al.	(16)	ESR, CRP, VAS, ODI	37.7 ± 25.4, 33.1 ± 29.3, 6.82 ± 2.14, 35.72 ± 1.91	11.4 ± 6.3, NA, 2.66 ± 1.04, 8.82 ± 1.73	26.3, NA, 4.16, 26.9	NA	NA	NA
Jia et al.	(10)	ESR, VAS, cobb angles	PLIF group: 38.5 ± 5.5, 9.1 ± 0.3, 33.0 ± 7.3/OLIF group: 38.3 ± 6.0, 9.1 ± 0.1, 33.5 ± 7.6	PLIF group: 5.3 ± 2.7, 0, 10.9 ± 2.8/OLIF group: 5.2 ± 2.5, 0, 11.1 ± 2.9	PLIF group: 33.2, 9.1, 22.3/OLIFgroup: 33.1, 9.1, 22.4	ASIA	PLIF group: 7C, 11D, 22E/OLIF group: 8C, 11D, 21E	PLIF group: 1D, 39E/OLIF group: 2C, 7D, 31E
Zhang et al.	(6)	ESR, CRP, VAS, ODI	35.5, 20.3 ± 10.2, 8.0, 88.5 ± 5.6	9.2 ± 3.6, 3.5 ± 1.7, 0, 9.3 ± 5.7	26.3, 16.7, 8.0, 79.2	ASIA	1B, 2C, 7D, 6E	16E
Zhang et al.	(8)	ESR, CRP, VAS, JOA, NDI	35.5 ± 20.6, 58.56 ± 44.42, 5.7 ± 1.6, 12.7 ± 3.7, 25 ± 8.5	6.7 ± 2.9, 5.57 ± 1.56, 0.4 ± 0.5, 16.4 ± 1.1, 3.7 ± 1.2	28.8, 52.99, 5.3, 3.7, 21.3	NA	NA	NA
Jiang et al.	(12)	ESR, CRP, VAS, ODI, JOA	Posterior group: 64.4 ± 28.4, 21.9 ± 19.6, 7.81 ± 1.0, 76.6 ± 2.3, 7.8 ± 1.4/anterior combined with posteriorgroup: 60.0 ± 28.4, 22.2 ± 19.4, 7.81 ± 1.0, 76.6 ± 2.3, 8.1 ± 1.5	Posterior group: 12.2 ± 4.1, 7.7 ± 3.2, 0.97 ± 0.8, 4.1 ± 2.0, 25.9 ± 0.9/anterior combined with posterior group: 11.9 ± 4.6, 7.6 ± 3.2, 0.97 ± 0.9, 4.0 ± 2.4, 26.2 ± 0.9	Posterior group: 52.2, 14.2, 6.84, 72.5, 18.1/anterior combined with posterior group: 48.1, 14.6, 6.84, 72.6, 18.1	NA	NA	NA
Abulizi et al.	(13)	ESR, CRP, VAS, ODI, JOA	46.03 ± 12.73, 41.47 ± 41.74, 5.19 ± 1.47, 55.31 ± 9.16, 12.38 ± 2.98	8.86 ± 3.05, 4.56 ± 1.75, 0.47 ± 0.67, 10.72 ± 3.23, 26.13 ± 2.58	37.17, 36.91, 4.72, 44.59, 13.75	NA	NA	NA
Li et al.	(14)	ESR, CRP, VAS, JOA, cobb angles	Non-debridement group: 42.21 ± 29.87, 23.58 ± 20.99, 4.50 ± 1.26, 19.79 ± 2.47, 13.48 ± 2.28/debridement group: 44.09 ± 28.13, 33.96 ± 26.4, 4.82 ± 1.19, 18.72 ± 3.02, 13.63 ± 2.08	Non-debridement group: 5.90 ± 3.34, 1.25 ± 1.03, 1.14 ± 0.35, 27.48 ± 1.15, 5.63 ± 0.60/debridement group: 5.72 ± 3.53, 1.64 ± 1.52, 1.10 ± 0.31, 27.93 ± 0.99, 5.57 ± 0.62	Non-debridement group: 36.31, 22.33, 3.36, 7.69, 7.85/debridement group: 38.37, 32.32, 3.72, 9.21, 8.06	NA	NA	NA
Wang et al.	(17)	ESR, CRP, VAS, ODI	60.8 ± 27.1, 35.3 ± 30.6, 6.9 ± 0.9, 58.4 ± 13.0	7.4 ± 3.2, 4.7 ± 1.2, 0.6 ± 0.7, 8.0 ± 4.6	53.4, 30.6, 6.3, 50.4	ASIA	6D, 8E	2D, 12E
Luan et al.	(18)	ESR, CRP, VAS, ODI, JOA	41.35 ± 15.50, 33.61 ± 18.54, 6.04 ± 1.49, 54.08 ± 9.92, 15.12 ± 3.89	7.31 ± 2.34, 2.04 ± 0.71, 0.72 ± 0.53, 10.44 ± 5.04, 25.43 ± 3.49	34.04, 31.57, 5.32, 43.64, 10.31	ASIA	3C, 17D, 25E	2D, 53E
Yin et al.	(20)	VAS	7.1 ± 2.9	0.8 ± 3.4	4.16	ASIA	1B, 4C, 5D, 2E	2D, 14E
Chen et al.	(19)	ESR, CRP, VAS	60.8 ± 22.2, 62.2 ± 39.9, 7.5 ± 1.4	NA, NA, 0.8 ± 0.7	NA, NA, 6.7	ASIA	6C, 10D, 8E	2D, 22E
Zhao et al.	(4)	VAS	5.85 ± 1.26	1.69 ± 1.35	4.16	ASIA	10C, 13D, 38E	4D, 57E
Na et al.	(21)	VAS, cobb angles	Anterior group: 7.1 ± 1.2, 14.6 ± 1.2/Posterior group: 6.9 ± 0.9, 15.4 ± 1.8	Anterior group: 1.2 ± 0.8, 7.7 ± 1.5/Posterior group: 1.1 ± 0.9, 6.6 ± 0.9	Anterior group:5.9, 6.9/Posterior group: 5.8, 8.8	NA	NA	NA
Liu et al.	(9)	ESR, CRP, VAS, JOA, ODI	ULIF group: 44.13 ± 22.13, 42.93 ± 15.31, 6.60 ± 1.45, 10.47 ± 3.52, 63.73 ± 14.13/open group: 46.76 ± 17.79, 44.35 ± 14.73, 6.24 ± 1.75, 11.18 ± 2.98, 61.65 ± 13.07	ULIF group: 4.13 ± 2.45, 3.47 ± 1.60, 0.27 ± 0.46, 27.33 ± 1.29, 6.93 ± 1.62/open group: 4.53 ± 2.65, 3.71 ± 1.36, 0.35 ± 0.49, 27.18 ± 1.19, 6.65 ± 1.77	ULIF group: 40, 39.46, 6.33, 16.86, 56.8/open group: 42.23, 60.64, 5.89, 16, 55	NA	NA	NA
Yang et al.	(11)	ESR, VAS, cobb angles	Posterior group: 38.5 ± 5.6, 9.4 ± 0.8, 33.0 ± 7.1/anterior group: 38.3 ± 6.1, 9.4 ± 0.9, 33.7 ± 7.1	Posterior group: 8.3 ± 3.3, 0.1 ± 0.3, 11.3 ± 4.7/anterior group: 7.7 ± 2.9, 0.1 ± 0.3, 11.7 ± 4.9	Posterior group: 30.2, 9.3, 21.7/anterior group: 30.6, 9.3, 22	ASIA	Posterior group: 32C + D, 32E/anterior group: 42C + D, 28E	Posterior group: 1C, 14D, 63E/anterior group: 1C, 11D, 58E
Su et al.	(15)	ESR, CRP, VAS, ODI, cobb angles	TLIF group: 41.18 ± 20.08, 30.06 ± 21.58, 8.68 ± 0.8, 87.89 ± 4.59, 19.04 ± 4.59/OLIF group: 44.95 ± 17.29, 29.87 ± 16.90, 8.90 ± 0.72, 88.11 ± 4.28, 20.34 ± 4.11	TLIF group: 6.98 ± 2.45, 1.61 ± 0.83, 0.68 ± 0.63, 6.00 ± 2.51, 9.80 ± 2.77/OLIF group: 7.23 ± 2.61, 1.91 ± 0.99, 0.80 ± 0.62, 6.78 ± 2.74, 9.65 ± 2.37	TLIF group: 34.2, 28.45, 8, 81.89, 9.24/OLIF group: 37.72, 27.96, 8.1, 81.33, 10.69	ASIA	TLIF: 5B, 6C, 2D, 3E/anterior surgery combined with posterior group: 5B, 3C, 1D, 3E	TLIF: 1D, 15E/anterior surgery combined with posterior group: 3D, 9E

VAS, visual analog scale; ODI, oswestry disability index; JOA, Japanese orthopaedic association; ESR, erythrocyte sedimentation rate; CRP, C-reactive protein; NDI, neck disability index; TLIF, transforaminal lumbar interbody fusion; OLIF, oblique lateral interbody fusion; PLIF, posterior lumbar interbody fusion; ASIA, American spinal injury association; NA, not available.

### Spinal fusion results

With the exception of one study (10), all of the remaining studies reported overall fusion rates (Table 5), and it is noteworthy that 660 of 666 patients achieved a 1- or 2-level fusion, for an overall fusion rate of 99%, with 4 studies (7, 9, 13, 18) reporting good fusion rates (1-level fusion rates), and 117 of 130 patients achieved a 1-level fusion, for an good fusion rate of 90%. 8 studies (8, 10–12, 18–21) reported time to fusion, and the average fusion time for 427 patients was 6.9 (4.8–9) months. Six studies (4, 6–8, 12, 16) mentioned the use of an interbody fusion device, and all 189 patients achieved either a 1- or 2-level fusion, but did not report an excellent fusion rate for cage.

**Table 5:** Fusion effects and complications.

Study	Year	Spines at follow up, *n*	Spines with follow imaging, *n*	Imaging	Operation type	Patients with preoperative antibiotics, *n* (time, d); Postoperative antibiotics, *n* (time, mo)	Patients with debridement, *n*	Total fusion success, n; Total fusion rate;(grade I, *n*; grade II, *n*; good fusion rate)	Fusion time, mo	Cage, Y/N	Complications
Wang et al.	(7)	13	13	x-ray, CT, MRI	ULIF	13 (>14); 13 (>3)	13	13; 100% (12 grade I; 1 grade II; 92.31%)	NA	Y	1 superficial infection
Wang et al.	(16)	22	NA	NA	20 TLIF, 1 anterior combined with posterior surgery, 1 Revision surgery	22 (>21); 22 (>6)	22	22; 100% (NA; NA; NA)	NA	Y	NA
Jia et al.	(10)	80	NA	NA	40 PLIF, 40 OLIF	80 (14–21); 80 (NA)	80	NA	PLIF group: 8.8 ± 0.5 OLIF group: 9.0 ± 0.7	NA	PLIF group:4 absecss recurrence
Zhang et al.	(6)	16	16	x-ray, CT	Posterior surgery	16 (>42); 16 (>6)	16	16; 100% (NA; NA; NA)	NA	Y	NA
Zhang et al.	(8)	15	15	x-ray, CT	Anterior surgery	2 (15); 15 (6.1 ± 1.9)	15	15; 100% (NA; NA; NA)	4.8 ± 1.4	Y	1 bladder dysfunction, 1 limb dysfunction
Jiang et al.	(12)	62	62	NA	33 posterior surgey, 29 anterior combined with posterior surgery	62 (>7); 62 (>1.5)	62	62; 100% (NA; NA; NA)	Posterior group: 7.6 ± 0.8, anterior combined with posterior group: 7.3 ± 0.8	Y	2 superficial wound infection, 1 intraoperative peritoneal rupture, 1 postoperative ileus, 1 iliac vein injury
Abulizi et al.	(13)	32	32	x-ray, CT	TLIF	32 (>14); 32 (>3)	32	32; 100% (30 grade I; 2 grade II; 93.75%)	NA	N	1 superficial infection
Li et al.	(14)	63	63	NA	34 non-debridement surgery, 29 debridement surgery	63 (7–21); 63 (3–4)	Non-debridementgroup: 34, debridement group: 0	Non-debridement group: 31; 92%(NA; NA; NA), debridement group: 28; 96%(NA; NA; NA)	NA	N	Non-debridement group: 2, debridement group: 1
Wang et al.	(17)	14	14	x-ray, CT	OLIF	14 (14); 14 (>1.5)	14	22; 100% (NA; NA; NA)	NA	N	1 subsidence of autologous iliac bone, 1 wound infection
Luan et al.	(18)	55	55	NA	Posterior surgery	55 (NA); 55 (>1.5)	55	55; 100% (48 grade I; 7 grade II; 87.27%)	6.9 ± 0.7	N	NA
Yin et al.	(20)	16	16	NA	Anterior surgery	16 (NA); 16 (1.5)	16	16; 100% (NA; NA; NA)	4.8 ± 1.3	N	1 wound infection, 1 pain of graft harvesting site
Chen et al.	(19)	24	24	x-ray, CT	Posterior surgery	24 (14); 24 (6.5 ± 2.5)	24	24; 100% (NA; NA; NA)	6.8 + 1.6	NA	N
Zhao et al.	(4)	61	61	x-ray, CT	Posterior surgery	61 (NA); 61 (>6)	61	61; 100% (NA; NA; NA)	NA	Y	6 wound infection
Na et al.	(21)	27	27	NA	14 anterior surgery, 13 posterior surgery	27 (NA); 27 (>3)	27	27;100% (NA; NA; NA)	Anterior group: 7.9 ± 1.9 posterior group: 8.8 ± 1.4	NA	1 wound infection, 1 loosening of fixation
Liu et al.	(9)	32	32	x-ray, CT	15 ULIF, 17 posterior surgery	32 (NA); 32 (3)	32	ULIF group: 14; 93.33% (13 grade I; 1 grade II; 86.67%), posterior group: 16; 94.17% (14 grade I; 1 grade II, 82.35%)	NA	N	NA
Yang et al.	(11)	148	148	NA	78 posterior surgery, 70 anterior surgery	148 (9–21); 148 (NA)	148	148; 100% (NA; NA; NA)	Posterior group: 8.7 ± 0.3, anterior group: 8.6 ± 0.4	N	1 wound infection, 3 paravertebral abscess, 2 pneumothorax
Su et al.	(15)	28	28	NA	12 TLIF, 16 anterior surgery combined with posterior surgery	28 (14–21); 28 (3)	28	28; 100% (NA; NA; NA)	NA	N	1 lung infection, 1 pressure ulcer, 2 wound infection, 1 lower extremity deep vein thrombosis, 1 digestive discomfort

CT, computed tomography; MRI, magnetic resonance imaging; ULIF, unilateral biportal endoscopic lumbar interbody fusion; TLIF, transforaminal lumbar interbody fusion; OLIF, oblique lateral interbody fusion; PLIF, posterior lumbar interbody fusion; NA, not available; Y, yes; N, no.

### Complications

Complications were reported in twelve studies (4, 7, 8, 10–15, 17, 20, 21), with 36 postoperative complications out of a total of 442 patients, an incidence of 8% (Table 5). Of these, one study (14) did not report specific types of complications, with the most common complication being incision infection, which occurred in 15 out of a total of 34 patients with complications, or approximately 44.12%, 3 cases of paravertebral abscesses, 2 cases of pneumothorax, 1 case of bladder dysfunction, 1 case of limb dysfunction, 1 case of peritoneal rupture, 1 case of ileal obstruction, 1 case of iliac vein injury, 1 case of autologous iliac bone subsidence, 1 case of pain at the graft harvesting site, 1 case of loosening of the internal fixation due to osteoporosis, 1 case of pneumonia, 1 case of deep vein thrombosis in the lower limbs, and 1 case of digestive discomfort.

## Discussion

The incidence of brucellosis is very high, with more than 500,000 people worldwide suffering from brucellosis each year (1), and brucellosis spondylitis is one of the most common complications, accounting for approximately 2%–53% of cases (2). As Brucella spondylitis is a bacterial infection, it carries a high risk of fusion failure, because in the presence of bacterial infection, when the implant enters the body, bacteria can easily adhere to its surface and form a biofilm, which evades the action of the immune system and antibiotics, resulting in an infection that is difficult to be cleared completely and continues to persist, and when biofilm adheres to the endoprosthetic device, it may affect the stability of the endoprosthetic device, which may in turn It cannot well maintain the normal position and status of the fusion area in the postoperative recovery phase, and also interferes with the bone fusion, and when the biofilm is attached to the implant material or interbody fusion device, it may interfere with the apposition with the autogenous bone, leading to the failure of fusion (24). In addition, the toxins of bacteria, triggering an immune response may disrupt the bone metabolic balance, and when the osteoclastic response is stronger than the osteogenic response, fusion may also be affected (25). The inflammatory response produced by bacteria deteriorates the quality of the bone in the area of fusion, and the lack of a sufficiently stable, healthy bone structure to serve as the basis for fusion makes it difficult to achieve good bone fusion (26), and patients with bacterial infections tend to be immunocompromised and undernourished, which is also not conducive to spinal fusion. Combined with the high risk factors for fusion failure in bacterial spondylitis, there is a need to investigate the efficacy and prognosis of spinal fusion for Brucella spondylitis. Therefore, this study reviewed the clinical and imaging outcomes of spinal fusion in patients with Brucella spondylitis. We summarise data from all available evidence to better understand whether these patients with Brucella spondylitis are suitable for spinal fusion and whether fusion treatment for spondylitis leads to good structural and functional outcomes in such a cohort of Brucella spondylitis patients.

We found in this systematic evaluation that good outcomes can be achieved in patients with Brucella spondylitis. Notably, fusion was achieved in almost all patients (99%), with a 90% good fusion rate, which is lower than the 5%–50% failure rate for back surgery reported in the review (27). In addition, all postoperative scores were significantly higher than preoperative scores with few complications, and spinal fusion can achieve reliable pain and functional improvements in selected patients. zhao et al. (4) demonstrated that placement of an interbody fusion device for spinal fusion in patients with brucellosis spondylitis is safe and effective. Jia et al. (10) demonstrated that spinal fusion, regardless of access, is effective in removing lesions, relieving pain, maintains spinal stability, promotes implant fusion, and promotes inflammation control. Therefore, spinal fusion may be effective in patients with Brucella spondylitis who have failed to respond to conservative efforts and are experiencing neurological symptoms and spinal instability.

Given that there are many risk factors for bacterial lesions in the spine that influence the outcome of spinal fusion, how can we explain the fact that patients with Mycobacterium ruber spondylitis demonstrate better fusion outcomes? We can consider the change in the active volume of the bacterial lesions and the magnitude of their virulence. Firstly, an analysis of all the included studies revealed that perioperative antimicrobial therapy and/or debridement was performed in all of them, both of which can play a role in reducing the active volume of bacterial lesions, thus reducing the risk of fusion failure. Zhang et al. (8) reported that fusion was achieved in all of their patients even when preoperative antimicrobial therapy was administered to only 2 out of 15, suggesting that even if preoperative antimicrobial therapy is not used, only the combination of preoperative antimicrobial therapy and preoperative antimicrobial therapy can reduce the risk of spinal fusion failure. This suggests that even without preoperative antimicrobial therapy, only the combined use of debridement and postoperative antimicrobial therapy may have a better effect on reducing the active volume of bacterial lesions; Li et al. (14) compared the effects of spinal fusion between the debridement group and the undebrided group, and there was no significant difference in fusion effects between the two groups, which suggests that only perioperative antimicrobial therapy may have a better effect on reducing the active volume of lesions; Wang et al. (7) suggested that the scope of debridement should not be too large, or it may affect the implantation area. too much, otherwise the stability of the implanted area will be affected. Currently, the new effective methods for debridement mainly involve the application of minimally invasive surgery. Pola et al. (28) proposed that percutaneous transforaminal endoscopy or CT-guided abscess drainage may reduce trauma and is suitable for early intervention. Wang et al. (7) performed unilateral biportal endoscopic (UBE) surgery on 13 patients with BS, which included debridement of the infected focus in the intervertebral disc, bilateral decompression of the spinal canal, and implantation of an interbody fusion cage. Eventually, all patients achieved satisfactory clinical outcomes and met the criteria for postoperative clinical cure. Wang et al. (17) treated 14 patients who met the surgical indications with minimally invasive oblique lateral interbody fusion (Mis-OLIF) surgery, which included debridement and fusion. No lateral or posterior internal fixation was performed. During the final follow-up, all patients achieved spinal fusion and clinical improvement. Secondly, the invasive capability of Brucella less than that of Staphylococcus aureus and tuberculosis, and thus the impact of various risk factors may be less (29). This suggests to us that, in conjunction with perioperative antimicrobial agents and debridement, we can treat Brucella spondylitis, which is relatively less invasive, with spinal fusion.

Through long-term follow-up, patients with Brucella spondylitis who chose conservative treatment had a higher risk of spinal deformity and chronic pain than those who chose surgical treatment (30). In addition, patients with chronic low back pain associated with conservative treatment were at high risk of depression (31). Amin et al. (32) found that inflammation of the spine, if not treated in a timely manner or with the wrong approach, has the potential to lead to other complications such as myelitis, meningitis, and encephalitis, which can have a devastating effect on the patient's quality of life. Regarding the optimal timing of the surgery, Hadjipavlou et al. (33) suggest early surgery. They believe that early abscess drainage can shorten the course of the disease and reduce nerve damage. However, Koubaa et al. (34) suggest delayed surgery. They advocate for standardized anti-infection treatment first to avoid unnecessary surgical trauma. In general, the timing of the surgery needs to be evaluated on an individual basis, and the overall principle is: “Control the infection first, and then repair the spine.” Specifically, it mainly includes the following two points: (1) When there are no clear indications, antibacterial treatment should be carried out temporarily first. After the infection is controlled, a one-stage surgery can be performed for spinal instability and residual lesions. (2) For sudden and severe deterioration of nerve function, even paraplegia, the compression of the abscess can be relieved first, such as through abscess drainage. After a few weeks or months, when the infection is controlled, a two-stage surgery can be performed to restore spinal stability or remove residual lesions. In this study, 10 studies (4, 6, 11–13, 15, 18–21) reported the classification of one-stage and two-stage surgeries, but all of them were one-stage surgeries, which included a total of 469 patients. It is also worth noting that fusion was achieved in all cases in the end. There were 460 cases of grade I fusion, and the good fusion rate was 98.08%. Therefore surgical treatment is an important option for patients who meet the indications for surgery, and spinal fusion is important for patients who need it as a necessary option for surgical treatment.

The most important result was the favourable clinical outcome in patients with Brucella spondylitis after fusion surgery. Thus, although spinal fusion surgery reduces spinal motion by one segment, it provides an option for patients with Brucella spondylitis to preserve more spinal function with significant functional and clinical improvement.

This study still has some limitations. First, heterogeneity should not be underestimated. The interventions and objectives of the included studies were similar, but they differed in terms of population characteristics, lesion sites, laboratory tests, type of surgery (such as open or minimally invasive, anterior or posterior and so on), and outcome scores. Secondly the number of included studies was limited and the overall level of evidence was low, and more case-control design studies are needed to provide better evidence. In addition, these studies had different follow-up intervals, which may interfere with the observation of functional impact.

## Conclusion

Spinal fusion can achieve a high clinical success rate and has a favourable prognosis and pain relief in patients with Brucella spondylitis. Although patients with Brucella spondylitis have a number of high-risk factors affecting the outcome of fusion, in conjunction with medication and debridement, spinal fusion may be a good option with significant functional and clinical improvement.

## Data Availability

The original contributions presented in the study are included in the article/Supplementary Material, further inquiries can be directed to the corresponding author.
